# Omental Transposition in Treatment of Severe Ocular Surface Alkaline Burn: An Experimental Study

**Published:** 2014

**Authors:** Athar Shadmani, Kourosh Kazemi, Mohammad Reza Khalili, Masoomeh Eghtedari

**Affiliations:** 1 Poostchi Ophthalmology Research Center, Shiraz University of Medical Sciences, Shiraz, Iran; 2 Transplant Research Center, Nemazi Hospital, Shiraz University of Medical Sciences, Shiraz, Iran

**Keywords:** Chemical Burn, Omentum, Omental Pedicle, Omental Transposition, Stem Cell

## Abstract

Since alkaline substances can rapidly penetrate into the cornea and subsequently damage limbal stem cells, another source of stem cells may be necessary to reconstruct the ocular surface. Omentum has some such characteristics like ability to regenerate tissue as well as anti-inflammatory capacity. Presence of adult stem cells and pluripotent embryonic cell markers make it suitable in wound healing; therefore, it seems reasonable to evaluate whether omentum can be helpful to restoration of ocular surface in severe alkaline burn. In this experimental trial, two groups of dogs (5 in each) were assigned. Following ethics approval, ocular surface alkaline burn was induced in both groups by placing filter papers soaked with NaOH (0.5 mol/l) on the cornea of one eye. Subsequently, group 1 (n=5) was treated only by conventional therapy; group 2 (n=5) was treated with omental elongation and transposition to the injured eye immediately following injury. Both groups were followed for six months. Ocular surface was evaluated by slit lamp microscope and corneal clarity was assessed and graded. At the end of six months, corneal opacity and vascularization were significantly reduced in group 2 (p-values of 0.009, 0.049, and 0.032 for corneal opacity, fluorescein staining, and vascularization grades, respectively). We have concluded that transposition of omental pedicle may be an effective treatment for severe ocular surface alkaline burn although more studies might be required.

## INTRODUCTION

The transparency and avascularity of the self-renewing epithelium of the cornea rely on the existence and health of limbal stem cells. When entire corneal limbus is destroyed by chemical burn, the cornea is resurfaced by abnormal conjunctival epithelium, which results in chronic irritation and inflammation, persistent epithelial defects, stromal scarring, symblepharon, and finally thorough visual loss (1, 2). 

Since limbal epithelial stem cells are presented in limited numbers especially in cases with total bilateral limbal stem cell deficiency, use of extraocular sources of stem cells are of potential advantage (1-4). 

Omentum is a unique and versatile tissue to be found in the abdomen, which migrates to injured organs in the abdominal cavity and surrounds them to promote healing.

 Although its natural role is not clear, transposing omental pedicle to the injured organs (omental transposition) to promote healing process has been used in all over the human body (3) including reconstruction of thoracic wall (4), gastrointestinal anastomosis and repair (5), in urogenital procedures (5,6), brain and spinal cord injuries (7,8), ischemic cardiovascular (9), and neurological disorders (10, 11). 

On the other hand, chemical burn is an important and common type of eye injury (12) that is still a therapeutic challenge for ophthalmologists, especially in patients with bilateral damages.

We designed this experimental study aiming to evaluate the effects of omental transposition on the healing process of an injured eye in an animal model of severe ocular surface alkaline burn.

**Fig 1:**
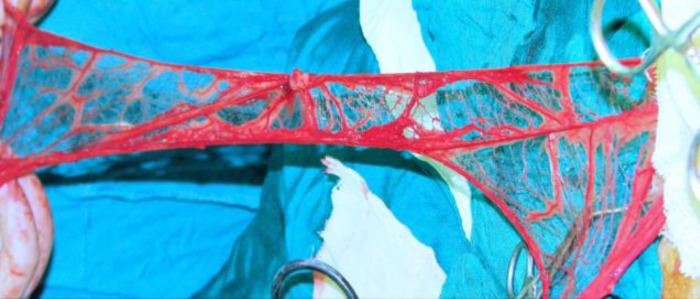
Elongated omentum is prepared and is ready to move to the injured eye.

**Fig 2:**
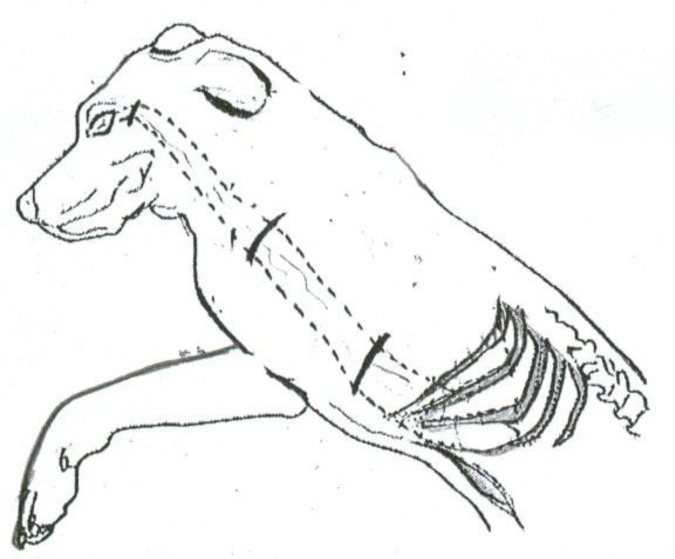
Transverse skin incisions and subcutaneous tunnel were made to move the elongated omentum to the surface of injured eye through the tunnel.

**Fig 3:**
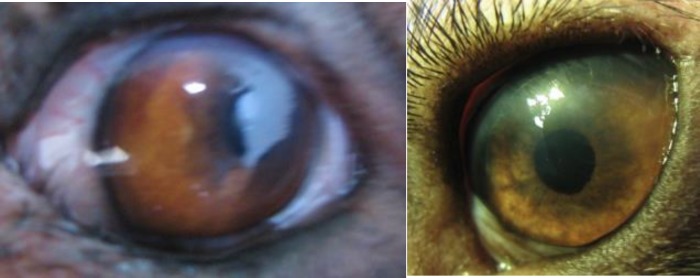
Eyes from group 2 (with omental transposition) at 6 months of follow-up. There is very little or no corneal opacity or vascularization.

**Fig 4:**
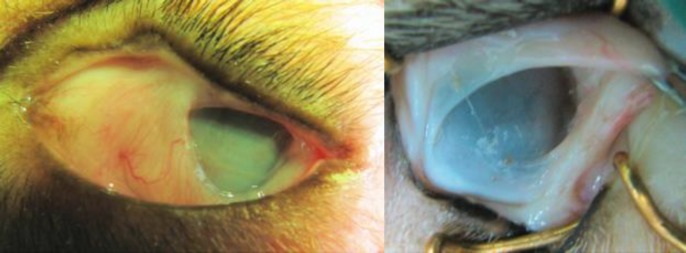
Eyes from Group 1 (without omental transposition) at six months of follow-up. There is severe symblepharon formation, corneal vascularization and opacity.

**Fig 5 (a,b):**
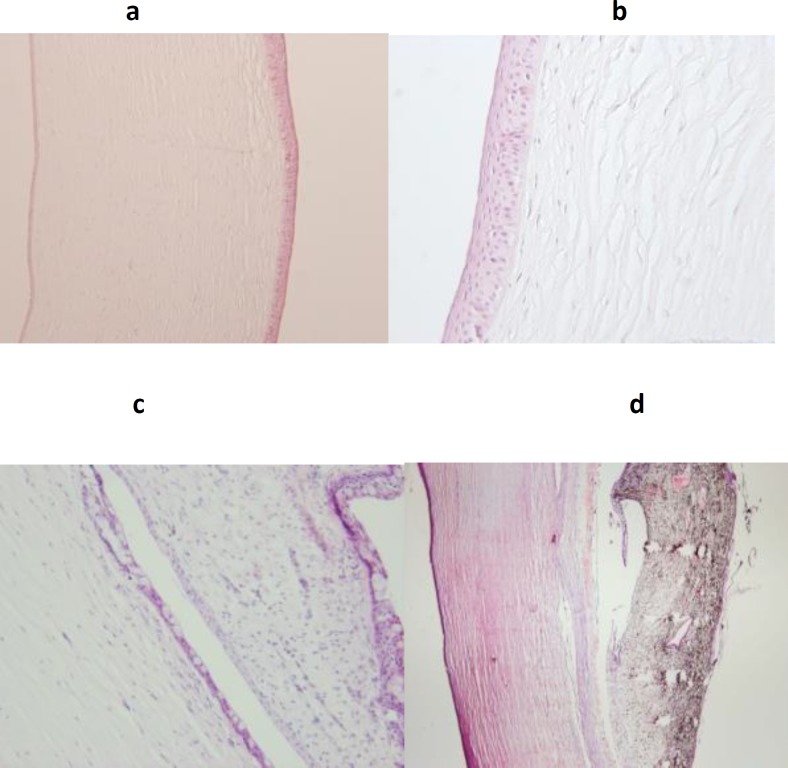
Pathologic slides from 2 cases with omental transposition: The corneal epithelium and stroma were fully reconstructed, with little evidence of acute or chronic inflammation. Two pathologic slides from cases without omental transposition. 5(c): cornea exhibited epithelial and stromal defects in variable degrees with moderate to severe conjunctivalization and numerous goblet cells on the corneal surface. 5 (d): there is corneal perforation with iridocorneal adhesion. (Hematoxillin & Eosin staining ; a,d:x100; b,c: x250. Note: Bowman's layer is normally absent in the cornea of dogs.

## MATERIALS AND METHODS

Ten adult male mongrel dogs (weighing 16-21 kg) were randomly divided into two groups. The animals were treated in accordance with National Institute's Guide for the Care and Use of Laboratory Animals and Guidelines of Animal Care and Use Committee of Shiraz University of Medical Sciences following to institutional ethical approval.

Group 1 consisted of five dogs in which grade 4 alkaline burn was created in one eye under anesthesia (according to Roper-Hall classification) (13). Filter papers (25 mm in diameter) soaked in 0.5 mol/l NaOH were placed on the cornea for this purpose. Then the ocular surface was rinsed with buffered saline until pH value regains its normal.

Cases in group 2 (n=5) were likewise pre-medicated with ketamine (10 mg/kg) followed by induction of anesthesia with 15 mg/kg of thiopental. After endotracheal intubation, halothane-oxygen mixture was used for maintenance of anesthesia. The preparation and stretching out of the omentum were performed by our team's general surgeon according to the procedure described by Goldsmith and colleagues (14) with some modifications. The vascular supply of omentum was preserved by the splenic artery, with especial attention to the importance of retaining of blood vessels as many as possible in the base of omental pedicle to maximize the chance of maintaining a vascular supply. When the omentum was stretched out (Fig. 1), transverse skin incisions were made cutting the chest wall, shoulder, and neck; approximately 4-5 cm wide with undermined margins. The openings were interconnected to form a subcutaneous tunnel, extending from the xiphoid area to the inferotemporal fornix of the desired eye. The stretched out omentum was tunneled to the surface of damaged eye (Fig. 2). Described manipulations were done attentively to avoid trauma to the third lid. The omental pedicle was laid on the conjunctiva and inferior limbus, yet secured by multiple 8-0 Vicryl sutures.

The laparotomy site and transverse skin incisions were closed properly. Following surgery, cases were observed until recovery from anesthesia. During the first four postoperative days, the operated animals received daily injection of 500 mg ceftriaxone and were fed by a routine diet.

 Following surgery, chloramphenicol eye drops were applied three times a day in both groups, and systemic analgesic was injected to the dogs in group 2. Detailed ophthalmologic examinations with slit lamp were done using Roper-Hall classification (13) once a week during the first month and monthly thereafter. The size of the epithelial defect was measured on the slit lamp using fluorescein vital staining. Measuring the area of defect by comparing it to total corneal surface scaled with a grid we made (we considered the corneal surface as a half sphere and used the best fitted 3X3 grid on it). The area of defect was reported as a fraction of 9. The corneal surface was examined for opacity and vascularization, and the results were recorded as percentile of total corneal surface. Symblepharon was graded considering the depth of upper and lower fornices.

All dogs were followed-up for six months. Results of various parameters were analyzed at week 2, 12 and 24. At the end of the study, enucleation of the damaged eyes was done under general anesthesia. The globes were fixed in 10% neutral buffered formaldehyde. Corneas, including 2-3 mm of limbal area were excised circumferentially and processed for hematoxylin and eosin staining (H&E) and histopathological examination was done with a light microscope.

Data were assessed for normality of distribution. Paired t-test was used to compare the differences in observed features between two groups. Data were presented as mean ±SD. P value of less than 0.05 was considered as statistically significant.

## RESULTS

Two weeks after the procedures, all of the damaged eyes in both groups showed 80-90% epithelial defect with a considerable amount of corneal opacity. There were no statistically significant differences between two groups on the vital fluorescein staining assessments. After 2-3 months, the corneas were significantly different in group 2 compared with control (group 1). Re-epithelialization, bookmarked with absence of even slightest fluorescein staining and significant corneal transparency, was observed in eyes of the dogs from group 2 (fig. 3). In group 1, the cornea failed to show noteworthy changes in clarity during the entire course of study. Persistent epithelial defect, corneal vascularization, stromal opacity, and ulcer formation in various degrees were seen in this group. Corneal perforation occurred in one eye of this group. Moderate to severe symblepharon was formed in four dogs of group 1 at the end of the study as shown in Fig. 4. Statistical analysis showed a significant difference between the two groups regarding the corneal opacity, fluorescein staining and vascularization grades at the end of six months (p-values were 0.009, 0.049, and 0.032 respectively). Mean fluorescent staining, corneal opacity, vascularization and symblepharon formation in both groups are presented in Table 1.

**Table1:** Flourescein Staining ,Corneal Opacity, Corneal Vascularization and Symblepharon Degrees in Both Groups (Mean± SD)

		**Group 1** **(n=1)**	**Group 2** **(n=5)**	**P value**
Fluorscein Staining	Two weeks	85.00±10	78.00±8	**0.27 ** b
	Three months	60.00±7	9.00±5	**0.007** a
	Six months	18.00±10	4.00±5	**0.049 ** a
Corneal Opacity	Two weeks	97.00±4	93.00±6.7	**0.309b**
	Three months	91.00±8	43.00±10	**0.009 ** a
	Six months	89.00±8	23.00±20	**0.009 ** a
Corneal Vascularization	Two weeks	7.00±4	10.00±3.5	**0.42 ** b
	Three months	45.00±11	15.00±5	**0.008 ** a
	Six months	44.00±12	19.00±12	**0.032 ** a
Symblepharon	Two weeks	6.00±8.9	1.00±2.2	**0.368 ** b
	Three months	52.00±25	16.00±18.1	**0.035 ** a
	Six months	55.00±27	16.00±18.1	**0.059 ** b

Group 1: without omental transposition. Group 2: with omental transposition.

a ) Significant P value.

b ) Insignificant P value

After six months, a tiny portion of tissue visible in the fornix of the treated eye was believed to be the remnant of pedicled omentum. Histopathology showed that the corneal epithelium and stroma were fully reconstructed in the group where omentum was transposed, with very few red blood cells in the superficial stroma in favor of fine fibrovascular pannus (Fig. 5a,b). In group 1, which served as the control, the corneas exhibited moderate to severe conjunctivalization characterized by presence of numerous goblet cells on the corneal surface as well as significant stromal vascularization (Fig. 5c,d). 

## DISCUSSION

Treatment of severe alkaline burn of the ocular surface is one of the puzzling challenges for the ophthalmologists, especially in bilateral cases. Our study was the one of the pioneer researches in the transpose of pedicled omentum to the injured eyes, suggesting a new technical procedure. Although we performed this procedure in a limited number of animals, the statistical analysis confirmed obvious differences between the two groups in all criteria. In human, omental elongation and transposition have been used all over the body from the brain (8) to the ankle (15). So, this technique can be considered for the eyes with severe alkaline burn and also in cases of limbal stem cell deficiency from other causes. Omentum has unique characteristics; for example, if it is exposed to foreign bodies, will expand rapidly to surround and encapsulate them. It increases in size and weight for more than 20 folds and also produces new interstitial cells and blood vessels (15). Adipocytes in the omentum counting for 70-95% of the total tissue in the un stimulated state but can be decreased to less than 30 % when exposed to foreign stimuli and the other 70% of the total omental mass is compensated by the stromal cells (16, 17). Activated omental tissue has abundant stromal cells, and when grown in culture media, expresses many interesting and unique groth factors. For instance, they express high level of vascular endothelial growth factor (VEGF). Furthermore, and most importantly, they express pluripotent cell markers (Oct–4 ( octamer–4), Nanog, SSEA–1( stage-specific embryonic antigen 1) and markers of adult stem cells (SDF–1α (stromal-cell-derived factor), CXCR4 ( chemokine receptor r 4), WT–1 (Wilms’ tumor antigen 1) (17), all may have effects in tissue repair.

The striking difference in corneal opacity and the stromal vascularization that was observed between the two groups in our study could be attributed to stem cells and other tissue repairing factors, derived from the pedicled omentum. We suggest omental stromal cells to be isolated and transferred from cadavers or the patient’s own omentum and even cultured in large quantities for this purpose. It can be a good way of treatment in cases with limbal stem cell deficiency. Obviously, there is a demand for further studies to evaluate the advantages and disadvantages of this procedure and its application on humans.
